# From Care Manager to Researcher: Addressing Health Disparities in Long-Term Services and Supports

**DOI:** 10.1093/geroni/igab046.1238

**Published:** 2021-12-17

**Authors:** Chanee Fabius

**Affiliations:** Johns Hopkins Bloomberg School of Public Health, Baltimore, Maryland, United States

## Abstract

Racial and socioeconomic disparities are prevalent in long-term services and supports (LTSS). There is a need for innovative research with practical application informing aging and disability policies to reduce health care disparities for older adults and people with disabilities using LTSS. This presentation will provide an overview of the career trajectory of Dr. Chanee Fabius, whose research agenda is informed by applied care management experience, where she helped older adults remain at home and delay the need for nursing home care. She will also present findings from work that (1) examines networks of care used by older adults (e.g., paid care and/or support from family and unpaid caregivers) and how they vary by race and socioeconomic status and (2) describes the effect of LTSS utilization on quality of life and health service utilization across diverse groups of older adults.

